# Elimination Diets, Not Food Selectivity, Are Associated with Reduced Nutritional Status in Children with Autism Spectrum Disorder

**DOI:** 10.3390/nu18122008

**Published:** 2026-06-20

**Authors:** Paula Grębska, Anna Fedorczak, Tomasz Pytrus, Anna Dębińska, Anna Kofla-Dłubacz

**Affiliations:** 1 II Department and Clinic of Pediatrics, Gastroenterology and Nutrition, Wroclaw Medical University, Marii Curie-Sklodowskiej 50-52, 50-369 Wroclaw, Poland; tomasz.pytrus@umw.edu.pl (T.P.); anna.kofla-dlubacz@umw.edu.pl (A.K.-D.); 2 Department of Endocrinology and Metabolic Diseases, Polish Mother’s Memorial Hospital-Research Institute, Rzgowska 281/289, 93-338 Lodz, Poland; anna.fedorczak@iczmp.edu.pl; 3 I Department and Clinic of Pediatrics and Allergology, Wrocław Medical University, Chałubińskiego 2a, 50-368 Wrocław, Poland; anna.debinska@umw.edu.pl

**Keywords:** pediatrics, autism spectrum disorder, malnutrition, elimination diet, anthropometry

## Abstract

**Background/Objectives:** Autism spectrum disorder (ASD) is a neurodevelopmental condition characterized by deficits in social communication, restricted and repetitive behaviors, and sensory processing abnormalities, including food selectivity. Due to the lack of effective causal therapies, alternative approaches such as dietary interventions are increasingly being explored. This study aimed to assess the impact of dietary factors on the nutritional status of children with ASD. **Methods:** A total of 103 children (75 with ASD and 28 controls) were included. Nutritional status was assessed using biochemical markers and standardized anthropometric measurements. Associations between nutritional status and dietary factors, particularly elimination diets implemented either on medical indications or in the absence of clinical justification, were analyzed. **Results:** ASD diagnosis was independently associated with lower height SDS (Standard Deviation Score). Food selectivity was significantly associated with growth patterns: children with food selectivity showed a higher prevalence of short stature compared with the control group (15.2% vs. 0%, *p* = 0.033). Children following elimination diets had significantly lower BMI SDS compared with those without dietary restrictions (−0.35 [−1.29 to 0.05] vs. −0.22 [−0.78 to 1.14], *p* = 0.046), although only 11.1% had medical indications for such interventions. Among non-supplemented participants, vitamin D deficiency was significantly more prevalent in the ASD group (84.6% vs. 33.3%, *p* < 0.001). **Conclusions:** Elimination diets were the only dietary factor associated with a clinically relevant reduction in BMI SDS in children with ASD. Food selectivity alone was not associated with impaired nutritional status. Most elimination diets were implemented without confirmed medical indications. These findings highlight the importance of evidence-based dietary management and routine vitamin D supplementation in this population.

## 1. Introduction

Autism Spectrum Disorder (ASD) is a neurodevelopmental condition with onset in early childhood, characterized by persistent deficits in social communication and social interaction, accompanied by restricted and repetitive patterns of behavior, interests, or activities. These symptoms are present from early development, are pervasive and enduring, and result in clinically significant impairment in social, educational, or occupational functioning. ASD is considered a spectrum condition due to substantial heterogeneity in symptom severity, functional abilities, and support needs across affected individuals. Diagnosis is based on standardized criteria defined in the Diagnostic and Statistical Manual of Mental Disorders (DSM-5-TR) and the International Classification of Diseases (ICD-11) [1,2].

The increasing recognition of ASD reflects growing attention to this condition. In addition to a true increase in prevalence, several factors have been proposed to contribute to this trend, including broader diagnostic definitions, changes in diagnostic criteria and screening tools, methodological differences in research, and increased public and professional awareness [3,4,5]. The prevalence of ASD among 8-year-old children in the United States increased from 6.7 per 1000 in 2000 to 27.6 per 1000 in 2020, corresponding to approximately 2.8% of the population [6,7,8]. A 2022 meta-analysis including European data (33,579 participants) reported an ASD prevalence of 1.4% [9], which is substantially higher than the approximately 0.2% prevalence reported in studies from the 1990s [10,11]. Consequently, there is growing interest in ASD-related symptoms and interventions.

Proper nutrition is a critical component of supporting the overall health and development of individuals with ASD, particularly given the frequent presence of eating difficulties associated with autistic traits. Food selectivity is commonly reported and often contributes to mealtime challenges and family conflict [12,13,14]. This selectivity has been associated with a preference for high-energy, ultra-processed foods and reduced consumption of nutrient-rich foods. A systematic review published in 2025 reported higher consumption of cereals (e.g., pasta and rice) and lower intake of fruits and vegetables among children with ASD, affecting both macronutrient and micronutrient intake [15,16,17].

Given the evidence that children with ASD often exhibit restricted dietary patterns and that nutritional status may influence health outcomes, nutritional interventions are increasingly recognized as an important component of ASD management [18,19,20]. Currently, no pharmacological procedures effectively address the core symptoms of ASD, and children with ASD may be more susceptible to the adverse effects of psychoactive medications compared with their neurotypical peers [21,22,23]. Therefore, alternative approaches are being explored.

Dietary interventions, including gluten-free and casein-free diets (GFCF), gluten-free diets (GFD), and ketogenic diets (KD), have been proposed as potential therapeutic strategies in ASD. These approaches are often believed to alleviate gastrointestinal and behavioral symptoms. A systematic review published in 2025 examining complementary and alternative medicine in ASD demonstrated a shift over the past decade toward interventions based on dietary modifications and nutraceuticals, including vitamins and minerals, omega-3 fatty acids, prebiotics, probiotics, and digestive enzymes. However, current evidence does not support the effectiveness of some commonly used dietary interventions, such as the GFCF diet [24,25]. Conversely, a 2022 meta-analysis reported a positive effect of dietary therapy on ASD symptoms [26]; however, these studies did not assess the impact of dietary interventions on the nutritional status of children.

Importantly, clinical guidelines from organizations such as the National Institute for Health and Care Excellence (NICE) and the American Academy of Pediatrics (AAP) do not recommend routine implementation of special diets for all children with ASD. Instead, dietary modifications are advised only in the presence of specific co-occurring conditions, such as celiac disease, food intolerances, or significant gastrointestinal symptoms [27]. Despite this, survey studies indicate that many parents independently introduce dietary interventions (e.g., gluten-free/casein-free diets), often guided by personal beliefs, or information from non-medical sources, including the internet or peer groups, rather than consulting medical professionals [28,29,30,31]. For example, a study conducted in the United Kingdom reported that over 80% of parents implemented some form of dietary modification in children with ASD [30]. These findings suggest that parental decisions regarding dietary interventions are frequently driven by subjective factors rather than evidence-based medical recommendations.

In light of the growing popularity of alternative dietary approaches, the present study aimed to compare the nutritional status-defined by anthropometric and biochemical parameters-of children with ASD and neurotypical children. Additionally, we assessed the relationship between nutritional status and dietary interventions implemented either on medical recommendation or based solely on parental decision-making.

## 2. Materials and Methods

### 2.1. Study Population

An observational, case–control, comparative study was conducted between 2021 and 2024 at the II Department and Clinic of Pediatrics, Gastroenterology and Nutrition in Wrocław, Poland. A total of 103 children of both sexes were enrolled. The study aimed to compare nutritional status—assessed using biochemical and standardized anthropometric measurements—between groups and to explore associations between these parameters and dietary factors, particularly elimination diets introduced either for medical indications or based solely on parental decisions.

The study included two groups of children:

The study group consisted of 75 children aged 2 to 17 years diagnosed with Autism Spectrum Disorder (ASD), with diagnosis established according to the Diagnostic and Statistical Manual of Mental Disorders (DSM-5) criteria [32]. Due to the marked heterogeneity of ASD and the challenges in achieving sample homogeneity, participants were stratified into subgroups based on historical ICD-10 diagnostic categories available in the medical records (childhood autism and Asperger syndrome) [33]. We deliberately retained the historical ICD-10 classification because of the substantial clinical heterogeneity within autism spectrum disorder and considered that this approach allowed for a more structured exploratory analysis of differences in dietary behaviors and nutritional status among participants with distinct clinical presentations. We acknowledge that Asperger syndrome is no longer recognized as a separate diagnosis in DSM-5 or ICD-11; therefore, these subgroup analyses should be considered exploratory.

The control group included 28 neurotypical children aged 4 to 17 years diagnosed with functional gastrointestinal disorders (e.g., functional dyspepsia, irritable bowel syndrome, and functional constipation after exclusion of organic disease. During recruitment, children following any elimination diet were excluded from the control group. This population was selected because the study was conducted in a tertiary pediatric gastroenterology center, where recruitment of completely healthy children was not feasible. The characteristics of both groups are presented in Table 1.

The purpose of the study was explained to all participants and their caregivers, and written informed consent was obtained from parents or legal guardians. The study protocol was approved by the Bioethics Committee of Wrocław Medical University, Poland (No. KB 408/2021), and conducted in accordance with the 1975 Declaration of Helsinki.

Inclusion criteria were:

1. age between 2 and 18 years
2. absence of organic diseases affecting growth and nutritional status (control group)
3. written informed consent from parents or legal guardians

Exclusion criteria included:

1. prematurity (<37 weeks of gestation)
2. pharmacological treatment affecting growth or body composition
3. endocrine disorders

### 2.2. Assessment of Nutritional Status

#### 2.2.1. Anthropometric Assessment

All anthropometric measurements were performed in accordance with standardized procedures recommended by the World Health Organization (WHO). Measurements were conducted in the morning by a trained healthcare professional.

Height was measured to the nearest 0.1 cm using a wall-mounted stadiometer, and body weight was recorded to the nearest 0.1 kg using a calibrated digital scale. Participants were barefoot and wore light clothing during measurements. Body mass index (BMI) was calculated using Quetelet’s formula (kg/m^2^).

Planned body composition analysis (body fat percentage, body fat mass) determined by bioelectrical impedance analysis (BIA) proved useless because the sensitivity and special needs of individuals with autism prevented the study from being conducted in a proper, standardized manner.

Standard deviation scores (SDS) for height and BMI were calculated using age- and sex-specific reference standards. Height-for-age and BMI-for-age percentiles were derived using WHO growth charts for children aged 0–5 years [34], OLA charts for children aged 5–7 years [35], and OLAF charts for those aged 7–18 years [36]. Detailed anthropometric data are presented in Table 1.

#### 2.2.2. Biochemical Markers of Nutritional Status

Nutritional status was further assessed using biochemical markers reflecting protein status, lipid metabolism, and micronutrient levels. The following parameters were analyzed: serum albumin, prealbumin, lipid profile (LDL cholesterol, HDL cholesterol, triglycerides, and total cholesterol), and 25-hydroxyvitamin D [37,38].

Blood samples were collected via venipuncture in a fasting state on the morning of hospital admission. Serum was obtained from clotted blood after 30 min of incubation at 25 °C, followed by centrifugation (15 min at 1500× *g*). All biochemical parameters were measured using standard clinical laboratory methods. Data were obtained from patients’ medical records.

Each result was categorized as within the reference range (N), below (↓), or above (↑) the laboratory norm.

### 2.3. Dietary Interventions

Information on dietary behaviors and dietary interventions was obtained from parents or caregivers during structured interviews.

Based on the interviews, the presence or absence of food selectivity was assessed by a pediatric gastroenterologist according to standardized criteria: a persistent pattern of limited food acceptance, characterized by a limited variety of foods consumed and/or refusal to eat specific foods, associated with sensory hypersensitivity to the texture, taste, smell, color, temperature, or appearance of food, described as including food refusal, a limited food repertoire, and repeated consumption of preferred foods [39,40].

#### 2.3.1. Medical Indications

The prevalence of medically indicated dietary interventions was assessed. Among children following elimination diets, those with clinically justified indications were identified, including:

- food allergies classified according to established immunological mechanisms: type I (immediate, IgE-mediated hypersensitivity) and type IV (delayed, T cell-mediated hypersensitivity) [41,42]. IgE-mediated food allergy was defined by positive results in a food-specific IgE panel, including cow’s milk proteins, wheat, egg, or combined milk and egg proteins. Non-IgE-mediated allergy was identified based on clinical symptoms (e.g., cutaneous manifestations, gastroesophageal reflux, gastrointestinal bleeding) in the absence of detectable food-specific IgE.
- celiac disease [43]
- other conditions, including food intolerances, eosinophilic esophagitis, and chronic gastrointestinal diseases [44,45,46]
- vitamin D_3_ supplementation, as a commonly recommended intervention supported by substantial scientific evidence. Information regarding vitamin D supplementation was collected as a binary variable (supplemented vs. non-supplemented). Detailed data regarding dosage, duration, and adherence were not available.

#### 2.3.2. Absence of Medical Indications

Elimination diets implemented without medical justification were also evaluated and classified as:

- diets resulting from food selectivity associated with ASD-related behavioral characteristics [47]
- parent-driven dietary interventions, often influenced by personal beliefs or external information sources regarding dietary approaches in ASD [48,49]

### 2.4. Statistical Analysis

Continuous variables were assessed for normality using visual inspection (histograms and Q-Q plots) and the Shapiro–Wilk test. Depending on data distribution, comparisons between two independent groups were performed using Student’s *t*-test or the Mann–Whitney *U* test. Categorical variables were compared using the chi-square test.

Correlations between continuous variables were evaluated using Pearson’s correlation coefficient. Continuous variables were presented as median and interquartile range (median (Q1–Q3)), while categorical variables were expressed as counts and percentages (N (%)).

To assess independent associations, logistic regression models adjusted for age and sex were applied. Results were expressed as odds ratios (ORs) with 95% confidence intervals (CI). A two-sided *p* value < 0.05 was considered statistically significant.

## 3. Results

### 3.1. Study Population

A total of 103 children were included in the analysis, comprising 75 participants with autism spectrum disorder (ASD) and 28 controls. The ASD group had a significantly higher proportion of males compared with controls (85.3% vs. 53.6%, *p* = 0.001) (Table 2).

**Table 2:** Baseline characteristics of children with ASD and control groups.

	ASD (n = 75)	Control (n = 28)	*p*-Value
Sex: male	64 (85.3%)	15 (53.6%)	0.001
Age (years)	6.4 [4.6–11.7]	11.1 [8.4–13.6]	0.001
Weight (kg)	22.3 [16.3–38.2]	37.9 [27.7–57.4]	<0.001
Height (cm)	120 [104–144]	149 [132–165.5]	<0.001

Among children with ASD, two subgroups were distinguished: childhood autism (*n* = 49) and Asperger syndrome (*n* = 26). The subgroups did not differ significantly in age (median 6.3 vs. 8.1 years, *p* = 0.15) (Table 3).

**Table 3:** Baseline characteristics of children with ASD—divided into subtypes.

ASD Type	Autism	Asperger	
	n = 49	n = 26	
Age (years)	6.3 [4.25–9.70]	8.1 [4.75–12.00]	0.15
Height (cm)	116 [102–134]	123 [108–147.2]	0.12

### 3.2. Assessment of Nutritional Status

#### 3.2.1. Anthropometric Assessment

In age- and sex-adjusted linear regression models, ASD diagnosis was independently associated with lower height compared with controls (β = −6.0 cm, 95% CI: −10.1 to −2.0; β = −0.76 SDS, 95% CI: −1.40 to −0.11; *p* = 0.004 and *p* = 0.022, respectively). No significant differences between groups were observed for BMI SDS after adjustment (Table 4).

Similarly, there were no significant differences between ASD and control groups in BMI categories, height categories, or the combined phenotype of short stature with underweight (all *p* > 0.16; Table 5).

Height SDS did not differ significantly between ASD subgroups (all *p* > 0.10). However, children with childhood autism had significantly higher BMI SDS compared with those with Asperger syndrome (median −0.13 vs. −0.71 SDS, *p* = 0.037). When BMI categories were analyzed using 2 × 2 comparisons (underweight vs. not, normal weight vs. not, overweight/obese vs. not), no statistically significant differences were found between ASD subgroups (all *p* > 0.05). Detailed anthropometric data are presented in Table 6.

#### 3.2.2. Biochemical Markers of Nutritional Status

Overall, biochemical markers did not differ significantly between ASD and control groups. However, when analyzed categorically, children with ASD were more likely to have low prealbumin levels compared with controls (27% vs. 0%, *p* = 0.002). No significant differences were observed for albumin, lipid profile parameters, or 25-hydroxyvitamin D status (Table 7).

Comparisons between ASD subtypes showed no significant differences in any biochemical parameters, including vitamin D, albumin, prealbumin, or lipid profile (all *p* > 0.10). These results are summarized in Table 8.

### 3.3. Dietary Interventions

Dietary eliminations were significantly more common in the ASD group compared with controls (36.0% vs. 7.1%, *p* = 0.003). In logistic regression analysis adjusted for age and sex, ASD was independently associated with a higher likelihood of any dietary elimination (OR = 6.84, 95% CI: 1.40–33.39, *p* = 0.017). Neither age nor sex significantly influenced this association.

Regarding specific eliminated food groups, children with ASD more frequently avoided dairy (24.0% vs. 0%) and gluten (16.0% vs. 0%), while lactose-free diets were reported in both groups at low frequencies (2.7% vs. 7.1%). Egg elimination and multiple combined eliminations were observed exclusively in the ASD group (Table 9).

Within ASD subtypes, dietary eliminations were more common in children with Asperger syndrome than in those with childhood autism (53.8% vs. 26.5%, *p* = 0.019) (Table 10).

#### 3.3.1. Medical Indications

No significant differences were observed between the ASD and control groups in the prevalence of allergy-related symptoms. Rates of gastroesophageal reflux disease (GERD) (18.7% vs. 32.1%, *p* = 0.12) and cutaneous manifestations (2.7% vs. 0%) were similar between groups. Gastrointestinal bleeding was not reported in either group. The overall prevalence of any allergy-related symptoms was comparable (41.3% vs. 42.9%, *p* = 0.89).

The prevalence of clinically diagnosed food allergy (5.3% vs. 7.1%, *p* = 0.73) and celiac disease (2.7% vs. 0%, *p* = 0.30) was low and did not differ significantly between groups. Likewise, no differences were observed in the frequency of positive food-specific IgE results or in the distribution of specific allergens.

After adjustment for age and sex, ASD was not associated with the presence of allergy-related symptoms (aOR = 0.89, 95% CI: 0.33–2.38), GERD (aOR = 0.72, 95% CI: 0.24–2.16), or positive IgE test results (aOR = 0.87, 95% CI: 0.23–3.27; *p* = 0.84). Due to the low frequency of specific eliminations and food-related diagnoses, adjusted analyses for these variables were not feasible. All results are presented in Table 11.

Within ASD subtypes, children with Asperger syndrome were more likely to report allergy-related symptoms compared with those with childhood autism (76.9% vs. 49.0%, *p* = 0.027). However, individual symptom categories and diagnoses (food allergy, celiac disease) did not differ significantly between subgroups (Table 12).

Vitamin D status did not differ significantly between ASD and control groups (*p* = 0.80). However, vitamin D supplementation was markedly more frequent among children with ASD (81.1% vs. 3.6%, *p* < 0.001).

Stratified analyses showed no differences in vitamin D levels among children who received supplementation (*p* = 1.00). Among children with ASD, supplementation was associated with a significantly lower prevalence of vitamin D deficiency (21.8% vs. 84.6%, *p* < 0.001). In contrast, this relationship could not be reliably assessed in the control group due to the very low rate of supplementation.

Among children not receiving supplementation, vitamin D deficiency was significantly more prevalent in the ASD group compared with controls (84.6% vs. 33.3%, *p* < 0.001). Detailed results are presented in Table 13.

#### 3.3.2. In the Absence of Medical Indications

##### Food Selectivity

Selective eating was significantly more common in children with ASD compared with controls (44.0% vs. 7.1%, *p* < 0.001). After adjustment for age and sex, ASD was associated with significantly higher odds of selective eating (OR = 9.57, 95% CI: 1.95–46.87, *p* = 0.005) (Table 14).

Within ASD subtypes, selective eating was more frequent in the Asperger subgroup than in children with childhood autism (53.8% vs. 26.5%, *p* = 0.019).

Within the ASD group, children with and without selective eating did not differ significantly in age, anthropometric parameters (height, height SDS, BMI SDS), or biochemical markers (all *p* > 0.18). The frequency of dietary eliminations and food-related diagnoses was also comparable (all *p* ≥ 0.29).

However, selective eating was associated with growth patterns: children classified as selective eaters had a higher prevalence of short stature compared with non-selective eaters (15.2% vs. 0%, *p* = 0.033). Additionally, vitamin D deficiency was more frequent among selective eaters (21.9% vs. 7.3%, *p* = 0.036).

No significant differences were observed between selective and non-selective eaters in BMI categories, albumin levels, lipid profile, or vitamin D supplementation (all *p* > 0.10) (Table 15).

##### Eliminated Diet According to Parental Choice

Dietary eliminations were more common in children with Asperger syndrome than in those with childhood autism (51.9% vs. 25.0%, *p* = 0.019). Selective eating was not significantly associated with the use of elimination diets (*p* = 0.06).

The prevalence of diagnosed food allergy and celiac disease remained low and comparable across groups.

Children following elimination diets had significantly lower BMI SDS compared with those not following such diets [−0.35 (−1.29 to 0.05) vs. −0.22 (−0.78 to 1.14), *p* = 0.046]. They also had higher vitamin D levels [31.2 (24.6–44.1) vs. 36.3 (31.5–49.2), *p* = 0.045].

Other anthropometric and biochemical parameters did not differ significantly between groups (all *p* > 0.05). No differences were observed in BMI SDS or height SDS distribution by elimination status (all *p* > 0.05).

Similarly, no significant differences were found in categorical outcomes for vitamin D, albumin, prealbumin, LDL cholesterol, total cholesterol, HDL cholesterol, or triglycerides between children with ASD following elimination diets and those without eliminations (all *p* > 0.05). These findings are summarized in Table 16 and Table 17.

## 4. Discussion

The present study evaluated the relationship between dietary behaviors and nutritional status in children with autism spectrum disorder, with particular emphasis on elimination diets and food selectivity. Several clinically relevant findings emerged.

First, children with ASD were characterized by significantly lower height SDS compared with controls, independent of age and sex. This finding is consistent with previous reports suggesting altered growth patterns in children with ASD, potentially related to nutritional imbalances, feeding difficulties, or underlying metabolic and gastrointestinal factors [25,45]. Although BMI SDS did not differ between groups, the observed reduction in linear growth may indicate subtle chronic nutritional inadequacies that are not fully reflected by BMI alone.

Second, food selectivity was significantly more prevalent in children with ASD and was associated with specific growth patterns, particularly a higher prevalence of short stature among children with food selectivity. However, selective eating was not associated with lower BMI SDS or significant biochemical disturbances. These findings suggest that although food selectivity is a common behavioral feature of ASD, its direct impact on overall nutritional status may be less pronounced than previously assumed. This is consistent with previous studies indicating that children with ASD may maintain adequate caloric intake despite limited dietary variety, although micronutrient deficiencies may still occur [15,16,45].

Third, dietary elimination was significantly more frequent in children with ASD and was associated with lower BMI SDS. Importantly, elimination diets were the only dietary factor associated with indicators of reduced nutritional status in this cohort. This finding highlights the potential risk of inappropriate dietary restrictions, particularly when implemented without adequate medical supervision. Similar concerns have been raised in previous studies, which suggest that restrictive diets, especially gluten-free and casein-free diets, may lead to insufficient intake of essential nutrients and impaired growth when not properly managed [30,31,47].

Notably, the vast majority of elimination diets in this study were implemented in the absence of confirmed medical indications. The prevalence of food allergies, celiac disease, and other clinically justified conditions was low and comparable between the ASD and control groups. Furthermore, no association was found between allergy-related symptoms or IgE sensitization and the use of elimination diets. These findings strongly suggest that most dietary restrictions were parent-driven rather than medically indicated. This observation is consistent with previous reports demonstrating that parental beliefs and non-medical information sources play a significant role in dietary decision-making in ASD [28,29].

Differences between ASD subtypes were also observed. Children with Asperger syndrome were more likely to follow elimination diets and exhibit selective eating behaviors compared with those with childhood autism. They also more frequently presented with allergy-related symptoms, although these were not confirmed by objective diagnostic measures. This may reflect differences in parental perception, communication abilities, or behavioral profiles between ASD subtypes, potentially influencing dietary practices. These findings should be interpreted cautiously because the distinction between childhood autism and Asperger syndrome reflects historical diagnostic classifications and does not correspond to current DSM-5 or ICD-11 diagnostic criteria. Nevertheless, the historical ICD-10 classification was retained because of the marked heterogeneity of autism spectrum disorder and enabled a more structured exploratory assessment of potential differences in dietary behaviors and nutritional status between participants with distinct clinical presentations.

Biochemical markers of nutritional status were largely comparable between groups, with the exception of a higher prevalence of low prealbumin levels in children with ASD. This may indicate subtle differences in protein status or acute-phase responses. Prealbumin is a negative acute-phase reactant, and reduced concentrations may reflect inflammatory processes in addition to nutritional status. However, none of the participants included in this study presented elevated CRP concentrations at admission, suggesting that overt systemic inflammation was unlikely to account for the observed differences. Nevertheless, prealbumin should be interpreted cautiously, as factors other than nutritional status may affect its concentration. The clinical relevance of this finding requires further investigation.

Overall, the lack of major biochemical abnormalities suggests that overt malnutrition was not common in the studied population, despite differences in dietary behaviors [25].

An important finding of this study relates to vitamin D status. Although overall vitamin D status did not differ significantly between groups, this finding likely reflects the markedly higher supplementation rate among children with ASD. Therefore, the results should primarily be interpreted as demonstrating the effectiveness of supplementation in preventing deficiency rather than indicating similar underlying vitamin D status between ASD and control groups. In contrast, among non-supplementing children, vitamin D deficiency was substantially more common in the ASD group. These findings support the importance of routine vitamin D supplementation in children with ASD, particularly given their increased risk of dietary limitations and reduced sun exposure [25,46].

Taken together, the results of this study indicate that elimination diets, rather than food selectivity itself, represent the primary dietary factor associated with impaired nutritional status in children with ASD. Importantly, these dietary restrictions are frequently implemented without clear medical justification, raising concerns about their safety and long-term consequences. However, because of the cross-sectional design of the study, it cannot be determined whether elimination diets contributed to poorer nutritional status or whether children perceived as nutritionally vulnerable were more likely to be placed on elimination diets. Therefore, the observed associations should not be interpreted as evidence of causality.

This study has several limitations. The relatively small sample size may limit the generalizability of the findings and reduce statistical power for subgroup analyses. In addition, the ASD group was substantially younger than the control group. Although regression analyses were adjusted for age and sex, residual confounding cannot be completely excluded, particularly for anthropometric and biochemical outcomes.

Additionally, the case–control design may limit generalizability and preclude causal inference. Furthermore, the control group consisted of children with functional gastrointestinal disorders rather than completely healthy children. Although organic disease was excluded and participants following elimination diets were not included in the control group, gastrointestinal symptoms themselves may influence dietary behaviors and nutritional status. Therefore, this choice may have affected the magnitude of the observed differences between groups. The assessment of dietary behaviors was partly based on parental reporting, which may introduce reporting bias. Finally, detailed micronutrient intake was not evaluated.

Given the number of statistical comparisons performed, the possibility of type I error cannot be excluded. Therefore, statistically significant findings should be interpreted cautiously and confirmed in future studies.

Future studies should focus on larger cohorts, longitudinal designs, and comprehensive dietary assessments to better understand the long-term effects of dietary interventions in ASD. There is also a need for clear, evidence-based dietary guidelines to support clinicians and families in making informed decisions and to prevent unnecessary dietary restrictions.

## 5. Conclusions

In this cohort, elimination diets were the only dietary factor associated with lower BMI SDS in children with ASD. Importantly, most dietary eliminations were implemented in the absence of confirmed medical indications, suggesting that these practices are predominantly parent-driven rather than clinically justified. In contrast, food selectivity, although highly prevalent in ASD, was not associated with impaired anthropometric or biochemical nutritional status.

Children with ASD were characterized by lower height SDS compared with controls, indicating potential alterations in growth patterns. Additionally, vitamin D deficiency was markedly more frequent among non-supplementing children with ASD, while supplementation was associated with improved vitamin D status, supporting its routine use in this population.

These findings highlight the need for careful, evidence-based dietary management in children with ASD and support cautious evaluation of elimination diets, particularly when implemented without clear medical indications.

The limited number of studies addressing the relationship between nutrition and autism, despite the high prevalence of eating-related difficulties in this population, reflects a significant gap in current clinical knowledge. Despite the limitations of the present study, our findings provide clinically relevant insights into the nutritional challenges associated with ASD.

Further large-scale, longitudinal studies are required to better define the role of dietary interventions in ASD and to support the development of clear clinical guidelines aimed at preventing both malnutrition and unnecessary dietary restrictions.

## Figures and Tables

**Table 1:** Intervals of the Z-scores.

Classification	Z-Score Values
Weight-for-age z-score	
Low weight	Z < −2 SD
Normal weight	−2 SD < Z < +2 SD
Overweight	Z > +2 SD
Height-for-age z-score	
Short stature	Z < −2 SD
Normal stature	−2 SD < Z < +2 SD
Tall stature	Z > +2 SD
BMI-for-age z-score	
Underweight	Z < −1.64 SD
Normal weight	−1.64 SD < Z < +1 SD
Overweight	+1 SD < Z < +1.64 SD
Obesity	Z > +1.64 SD

**Table 4:** Association between ASD diagnosis and anthropometric parameters in children—regression models adjusted for age and sex.

Variable	β (ASD vs. Controls)	95% CI	*p*-Value
Height SDS	−0.76	−1.40 to −0.11	0.022
BMI SDS	0.09	−0.54 to 0.72	0.779
log body weight	−0.08	−0.21 to 0.06	0.253
log BMI	0.02	−0.08 to 0.12	0.688

continuous variables: median [Q1–Q3]; categorical variables: n (%).

**Table 5:** Anthropometric characteristics in the ASD vs. the control group.

Category	ASD (n = 75)	Control (n = 28)	*p*-Value	
BMI SDS	−0.23 [−0.99 to 0.78]	−0.13 [−0.83 to 0.92]	0.83	
Height SDS	−0.43 [−1.30 to 0.20]	0.17 [−0.78 to 1.07]	0.023	
Category	ASD (n = 75)	Control (n = 28)	Total (n = 103)	*p*-value
BMI categories				
Underweight	12 (16.0%)	1 (3.6%)	13 (12.6%)	0.18
Normal weight	48 (64.0%)	23 (82.1%)	71 (68.9%)	0.13
Overweight/obesity	15 (20.0%)	4 (14.3%)	19 (18.4%)	0.7
Height categories				
Short stature	5 (6.7%)	0 (0.0%)	5 (4.9%)	0.16
Normal stature	61 (81.3%)	24 (85.7%)	85 (82.5%)	0.49
High stature	9 (12.0%)	4 (14.3%)	13 (12.6%)	0.79
Combined phenotype				
BMI and Height < 3c	2 (2.7%)	0 (0.0%)	2 (1.9%)	0.38

**Table 6:** Comparison of anthropometric characteristics between ASD-autism and Asperger subgroups.

Category	Autism	Asperger	*p*-Value
Height SDS	−0.34 [−1.30–0.20]	−0.54 [−1.25–0.18]	0.88
BMI SDS	−0.13 [−0.76–1.10]	−0.71 [−1.22–0.07]	0.047
BMI categories			
Underweight	5 (10.2%)	7 (26.9%)	0.07
Normal weight	33 (67.3%)	15 (57.7%)	0.56
Overweight/obesity	11 (22.4%)	4 (15.4%)	0.56

**Table 7:** Biochemical characteristics in the ASD vs. the control group (laboratory norm (N), below (↓) and above this norm (↑).

		ASD (n = 75)	Control (n = 28)	*p*-Value
Protein status				
Albumin	N	44 (68.8%)	23 (82.1%)	0.18
	↓	20 (31.2%)	5 (17.9%)	
Prealbumin	N	54 (73%)	28 (100%)	0.002
	↓	20 (27%)	0 (0%)	
Lipid metabolism				
Total Cholesterol	N	49 (67.1%)	19 (67.9%)	0.94
	↑	24 (32.9%)	9 (32.1%)	
LDL	N	54 (79.4%)	21 (75%)	0.64
	↑	14 (20.6%)	7 (25%)	
HDL	↓	6 (8.8%)	1 (3.6%)	0.67
	N	62 (91.2%)	27 (96.4%)	
Triglycerides	N	12 (16.9%)	5 (17.9%)	0.91
	↑	59 (83.1%)	23 (82.1%)	

continuous variables: median [Q1–Q3]; categorical variables: n (%).

**Table 8:** Biochemical characteristics in subtypes od ASD (laboratory norm (N), below (↓) and above this norm (↑).

		Autism (n = 49)	Asperger (n = 26)	*p*-Value
Protein status				
Albumin	N	34 (70.8%)	20 (76.9%)	
	↓	14 (29.2%)	6 (23.1%)	0.63
Prealbumin	N	23 (60.5%)	21 (80.8%)	
	↓	15 (39.5%)	5 (19.2%)	0.07
Lipid metabolism				
Total Cholesterol	N	29 (61.7%)	20 (76.9%)	0.19
	↑	18 (38.3%)	6 (23.1%)	
LDL	N	32 (74.4%)	22 (88%)	0.18
	↑	11 (25.6%)	3 (12%)	
HDL	↓	3 (7%)	3 (12%)	0.66
	N	40 (93%)	21 (88%)	
Triglycerides	N	37 (82.2%)	22 (84.6%)	0.8
	↑	8 (17.8%)	4 (15.4%)	

continuous variables: median [Q1–Q3]; categorical variables: n (%).

**Table 9:** Dietary interventions in ASD vs. Controls.

	ASD (n = 75)	Controls (n = 28)	*p*-Value
Any dietary elimination	27 (36.0%)	2 (7.1%)	0.004
Specific eliminations			
Only dairy	11 (14.7)	0 (0%)	0.03
Dairy total	18 (24.0%)	0 (0%)	0.004
Only gluten	6 (8.0%)	0 (0%)	0.14
Gluten total	12 (16.0%)	0 (0%)	0.02
Egg	1 (1.3%)	0 (0%)	1.0
Lactose	2 (2.7%)	2 (7.1%)	0.3
Combined eliminations	7 (9.3%)	0 (0%)	0.19
Dairy + gluten	5 (6.7%)	0 (0%)	0.32
Dairy + egg	1 (1.3%)	0 (0%)	1.0
Dairy + gluten + egg	1 (1.3%)	0 (0%)	1.0

**Table 10:** Dietary interventions in subgroups.

	ASD (n = 49)	Asperger (n = 26)	*p*-Value
Any dietary elimination	13 (26.5%)	14 (53.8%)	0.02
Dairy elimination	10 (20.4%)	8 (30.8%)	0.32
Gluten elimination	7 (14.3%)	5 (19.2%)	0.58

**Table 11:** Allergy symptoms, food-related diagnoses, food-specific IgE in ASD vs. Controls.

	ASD (n = 75)	Controls (n = 28)	*p*-Value
Allergy diagnosis	4 (5.3%)	2 (7.1%)	0.66
Celiac disease	2 (2.7%)	0 (0%)	1.0
Other conditions	0 (0%)	0 (0%)	
Allergy type I-positive IgEs			
any	10 (13.3%)	5 (17.9%)	0.56
cow’s milk	5 (6.7%)	1 (3.6%)	1.0
wheat	1 (1.3%)	0 (0%)	1.0
egg	3 (4%)	1 (3.6%)	1.0
milk +egg	1 (1.3%)	0 (0%)	1.0
Allergy type IV			
any symptoms	44 (58.7%)	16 (57.1%)	0.89
GERD	14 (18.7%)	9 (32.1%)	0.12
Cutaneous manifestation	2 (2.7%)	0 (0%)	1.0
GI bleeding	0 (0%)	0 (0%)	

**Table 12:** Allergy symptoms, food-related diagnoses within ASD groups.

	ASD (n = 49)	Asperger (n = 26)	*p*-Value
Any allergy-related symptoms	24 (49.0%)	20 (76.9%)	0.027
GERD	7 (14.3%)	7 (26.9%)	0.17
Cutaneous manifestation	1 (2.0%)	1 (3.9%)	1.00
Allergy diagnosis	2 (4.1%)	2 (7.7%)	0.71
Celiac disease	1 (2.0%)	1 (3.8%)	0.71

**Table 13:** Vitamin D status and supplementation in ASD and control groups (laboratory norm (N), below (↓).

	ASD	Control	*p*-Value
Vitamin D status			
↓	24 (34.8%)	9 (32.1%)	0.8
N	45 (65.2%)	19 (67.9%)	
Vitamin D supplementation			
Yes	60 (81.1%)	1 (3.6%)	<0.001
No	14 (18.9%)	27 (96.4%)	
Vitamin D in supplementation group			
↓	12 (21.8%)	0 (0%)	1.0
N	43 (78.2%)	1 (100%)	
Vitamin D in non- supplementation group			
↓	11 (84.6%)	9 (33.3%)	<0.001
N	2 (15.5%)	18 (66.7%)	

**Table 14:** Selective eating in the study group.

	ASD (n = 75)	Controls (n = 28)	*p*-Value
Selective eating	33 (44.0%)	2 (7.1%)	<0.001
	Autism (n = 49)	Asperger (n = 26)	*p*-value
	13 (26.5%)	14 (53.8%)	0.02

**Table 15:** Comparison of anthropometric and biochemical categories between selective and non-selective eaters in the ASD group (laboratory norm (N), below (↓) and above this norm (↑).

Marker	Category	Selective Eaters (n = 33)	Non-Selective Eaters (n = 42)	*p*-Value
BMI	underweight	3 (9.1%)	9 (21.4%)	0.35
	normal	23 (69.7%)	25 (59.5%)	
	overweight/obesity	7 (21.2%)	8 (19.0%)	
Height	short stature	5 (15.2%)	0 (0%)	0.03
	normal stature	24 (72.7%)	37 (88.1%)	
	high stature	4 (12.1%)	5 (11.9%)	
BMI and Height < 3c	YES	2 (6.1%)	0 (0%)	0.11
	NO	31 (93.9%)	42 (100%)	
Prealbumin	↓	8 (29.6%)	12 (32.4%)	0.81
	N	19 (70.4%)	25 (67.6%)	
Albumin	↓	7 (21.9%)	13 (31%)	0.38
	N	25 (78.1%)	29 (69%)	
Total cholesterol	N	20 (62.5%)	29 (70.7%)	0.46
	↑	12 (37.5%)	12 (29.3%)	
LDL	N	23 (79.3%)	31 (79.5%)	0.99
	↑	6 (20.7%)	8 (20.5%)	
HDL	↓	28 (96.5%)	34 (87.2%)	0.23
	N	1 (3.5%)	3 (12.8%)	
Triglycerides	N	25 (80.6%)	34 (85%)	0.63
	↑	6 (19.4%)	6 (15%)	
Vitamin D3	↓	7 (21.9%)	17 (45.9%)	0.036
	N	25 (78.1%)	20 (54.1%)	
Supplementing D3	YES	26 (78.8%)	34 (82.9%)	0.65
	NO	7 (21.2%)	7 (17.7%)	

**Table 16:** Characteristics of children with ASD stratified by dietary eliminations.

Variable	Normal Diet (n = 48)	Elimination (n = 27)	*p*-Value
ASD subtype			
Childhood Autism	36 (75.0%)	13 (48.1%)	0.019
Asperger Syndrome	12 (25.0%)	14 (51.9%)	
Dietary interventions			
Selective eating	25 (52.1%)	8 (29.6%)	0.06
Allergy diagnosis	2 (4.2%)	2 (7.4%)	0.6
Celiac disease	1 (2.1%)	1 (3.7%)	1.0
Anthropometric Assessment			
Height SDS	−0.15 (−1.35 to 0.18)	−0.74 (−1.25 to 0.29)	0.60
BMI SDS	−0.22 (−0.78 to 1.14)	−0.35 (−1.29 to 0.05)	0.046
Biochemical Markers of Nutritional Status			
Albumin	4.60 g/dL (4.48–4.75)	4.50 g/dL (4.40–4.70)	0.27
Prealbumin	0.22 g/L(0.187–0.236)	0.21 g/L (0.183–0.266)	0.69
Triglycerides	70 mg/dL (45–99)	57 mg/dL (46–83)	0.44
LDL	88 mg/dL (78–105)	93 mg/dL (79–111)	0.62
Total cholesterol	157 mg/dL (146–180)	164 mg/dL (149–178)	0.61
HDL	53 mg/dL (45–63)	57 mg/dL (47–62)	0.46
Vitamin D	31.2 ng/mL (24.6–44.1)	36.3 ng/mL (31.5–49.2)	0.045
Vitamin D supplementation	35 (72.9%)	25 (92.6%)	0.055

**Table 17:** Comparison of anthropometric and biochemical categories in ASD children with and without elimination diet (laboratory norm (N), below (↓) and above this norm (↑).

	Normal Diet (n = 48)		Elimination (n = 27)	*p*-Value
BMI				
Underweight	5 (10.4%)		7 (25.9%)	0.15
Normal BMI	31 (64.6%)		17 (63.0%)	1.00
Overweight/obesity	12 (25.0%)		3 (11.1%)	0.25
Height				
Short stature	4 (8.3%)		1 (3.7%)	1.00
Normal stature	37 (77.1%)		24 (88.9%)	0.32
High stature	7 (14.6%)		2 (7.4%)	0.70
Other				
		Normal Diet (n = 48)	Elimination (n = 27)	*p*-Value
Protein status				
Albumin	N	36 (75%)	18 (69.2%)	
	↓	12 (25%)	8 (30.8%)	0.59
Prealbumin	N	27 (64.3%)	17 (77.3%)	
	↓	15 (35.7%)	5 (22.7%)	0.4
Lipid metabolism				
Total Cholesterol	N	31 (64.6%)	18 (72%)	0.52
	↑	17 (35.4%)	7 (28%)	
LDL	N	33 (75%)	21 (87.5%)	0.22
	↑	11 (25%)	3 (12.5%)	
HDL	↓	5 (11.4%)	1 (4.2%)	
	N	39 (88.6%)	23 (95.8%)	0.41
Triglycerides	N	38 (82.6%)	21 (84%)	1.00
	↑	8 (17.4%)	4 (16%)	

## Data Availability

The data presented in this study are available upon reasonable request from the corresponding author. The data are not publicly available due to privacy and ethical restrictions.

## Abbreviations

The following abbreviations are used in this manuscript:

ASD	Autism Spectrum Disorder
CAM	Complementary and Alternative Medicine
GFCF	Gluten Free Casein Free diet
NICE	National Institute for Health and Care Excellence
AAP	The American Academy of Pediatrics
